# hsa_circ_001946 elevates HOXA10 expression and promotes the development of endometrial receptivity via sponging miR-135b

**DOI:** 10.1186/s13000-021-01104-4

**Published:** 2021-05-16

**Authors:** Fang Zhao, Yihong Guo, Zhanrong Shi, Menglan Wu, Yuzhen Lv, Wenyue Song

**Affiliations:** 1 Department of Reproductive Medical Center, the First Affiliated Hospital of Zhengzhou University, Νo. 1 Jianshe East Road, Henan 450000 Zhengzhou, PR China; 2 Department of Reproductive Medical Center, Jiaozuo Maternity and Child Health Hospital, Jiaozuo, China

**Keywords:** hsa_circ_001946, HOXA10, miR-135b, Endometrial receptivity

## Abstract

**Background:**

Impaired endometrial receptivity is a major reason for embryo implantation failure. There’s a paucity of information regarding the role of circRNAs on endometrial receptivity. Here, we investigated the function of hsa_circ_001946 on endometrial receptivity and its mechanisms.

**Methods:**

A total of 50 women composing 25 with recurrent implantation failure and 25 who conceived after their implantation were recruited in this study. Expression of hsa_circ_001946, miR-135b, and HOXA10 was evaluated by quantitative RT-PCR (qRT-PCR) in biopsied endometrial tissue samples. The levels of HOXA10, and cell cycle markers (CCNB1, CDK1, and CCND1) were determined by IHC and western blotting assays. Binding relationship among miR-135b, hsa_circ_001946 and HOXA10 were confirmed by dual luciferase reporter assays and western blotting. MTT assays and cell cycle assays by FACS were employed to evaluate the proliferation and cell cycle of cells. T-HESCs were cultured with 1 µM medroxyprogesterone acetate (MPA) and 0.5 mM 8-bromoadenosine 3’:5’-cyclic monophosphate (8-Br-cAMP) to induce decidualization. The mechanisms and functions of hsa_circ_001946 on decidualization were further assessed by qRT-PCR evaluating the expression of hsa_circ_001946, miR-135b, HOXA10 and decidual markers (PRL and IGFBP1) in T-HESCs.

**Results:**

Endometrial tissues from patients with recurrent implantation failure had lower hsa_circ_001946 expression, higher miR-135b expression, and lower HOXA10 expression. Hsa_circ_001946 promoted HOXA10 expression by sponging miR-135b in T-HESCs. Overexpression of hsa_circ_001946 restored cell proliferation and cell cycle that were disrupted by miR-135b overexpression in T-HESCs. Decidualized T-HESCs had higher hsa_circ_001946 expression, lower miR-135b expression, and higher HOXA10 expression. Overexpression of hsa_circ_001946 reversed the expression of decidual markers (PRL and IGFBP1) that were suppressed by miR-135b overexpression in T-HESCs.

**Conclusions:**

In conclusion, our findings suggest that hsa_circ_001946 promotes cell proliferation and cell cycle process and increases expression of decidualization markers to enhance endometrial receptivity progression via sponging miR-135b and elevating HOXA10.

## Background

Infertility has been a highly prevalent condition worldwide [1]. *In vitro* fertilization-embryo transfer is an efficacious therapy for infertility; however, a number of patients still suffer implantation failure even after several *in vitro* fertilization attempts [2]. Endometrial receptivity is a major limiting factor for successful implantation, and insufficient endometrial receptivity is responsible for approximately 60 % of the implantation failures in women undergoing assisted reproduction [3, 4]. To transit into receptive state, the endometrium needs to undergo a series morphologically and functionally remodeling, during which stromal cells are hyper-proliferated, compacted, and differentiated into highly specialized cells with a secretory function (decidualization) [4]. The status of endometrium receptivity is associated with the expression of a range of genes like transcription factors, and cytokines and growth factors [5–7]. Any of which goes awry leads to the impaired receptivity; however, the mechanisms by which these factors mediate the endometrial receptivity remain poorly understood.

HomeoboxA-10 (HOXA10) is a homeobox-containing transcriptional factor that involves in regulating multiple receptive related genes; therefore, HOXA10 is considered as a key regulator for endowing the receptivity to endometrium [5, 8]. In endometrium, HOXA10 promotes the stromal cell proliferation, and increase in HoxA10 expression is necessary to initiate the transformation from endometrial stromal cells to secretory cells (decidualization) which is a foundation for the receptive endometrial microenvironment [5, 9]. Clinically, abnormal decrease of HoxA10 expression results in the failure of endometrial receptivity and implantation during *in vitro* fertilization treatment [10, 11]. Researchers reported that loss of HoxA10 impedes stromal cell proliferation during the process of decidualization via arresting the cell cycle [8, 12]. However, the mechanisms responsible for the regulation of HoxA10 expression in human endometrium are still elusive.

In recent years, a growing number of studies suggest that miR-135b is a regulator of HOXA10 in endometrial receptivity and embryo implantation. Expression of miR-135b was higher, while expression of HoxA10 was lower in endometrial tissues of infertile women compared to the control [7]. Similarly, the expression of miR-135b was higher and inversely correlated with the expression of HOXA10 in endometrial cells from patients with endometriosis [6]. These suggest that increase in miR-135b expression correlates with a downregulation of HOXA10, impaired endometrial receptivity, and implantation failure, but the exact mechanism for this regulation is unclear. Circular RNAs (circRNAs) is a new class of endogenous non-coding RNAs and have been a research hotspot recently. CircRNAs are extremely rich in binding sites for microRNAs, and therefore can competitively bind to and sequester miRNAs, leading to decrease in functional miRNA molecules for target mRNAs [13]. Available evidence has suggested a role of circRNAs-miRNA interaction in a variety of diseases [14–18]. However, there’s a paucity of information regarding the role of circRNAs in endometrial receptivity.

In the present investigation, we investigated the effects and mechanism of hsa_circ_001946 on endometrial stromal cells biological function and development of decidualization.

## Materials and methods

### Human subjects and Sample Collection

This study was conducted at the Reproductive Center of the First Affiliated Hospital of Zhengzhou University. Women undergoing *in vitro* fertilization treatment were invited to participate in this study. A total of 50 women comprising 25 infertile subjects and 25 control subjects were enrolled in this study. The control subjects were women conceived after *in vitro* fertilization treatment (Control group). These infertile subjects were women with recurrent implantation failure (RIF group). Including criteria were women younger than 40 years, with no hydrosalpinx, no polycystic ovarian syndrome, no previous surgery for adenomyosis, no uterine malformations, no abnormal uterine bleeding, no endometrial abnormalities, and did not receive any hormone therapy.

During day 20 to 24 of the menstrual cycles, endometrium tissue was obtained by an endometrial biopsy using a sterile Pipelle curette (Pipelle de Cornier, France). After collection, the biopsied tissues were divided into two parts. One part was fixed in 10 % buffered formalin, transferred to 70 % ethanol, and then embedded in paraffin for immunohistochemical staining, and the other part was placed in a tube containing RNAlater® (Ambion; Thermo Fisher Scientific, Inc.) and stored in liquid nitrogen for further processing.

All experimental procedures related with human tissues were approved by the Ethical Review Committee of University of Zhengzhou. All patients have signed a paper version of informed consent before samples were taken.

### Immunohistochemical staining assays

Paraffin sections were deparaffinized, hydrated with an ethanol gradient, and boiled for antigen retrieval. The sections were blocked with 5 % goat serum in Tris-buffered saline, 0.05 % Tween 20 (TBST) (Sigma-Aldrich) for 1 h at room temperature. Then, the sections were incubated overnight at 4 °C with the primary antibody anti-HOXA10 (Abcam; ab191470). Then, the sections were incubated with incubated with secondary antibody HRP conjugated goat anti-rabbit antibody for 30 min and then positive expression of HOXA10 were visualized using a 3,3′-diaminobenzidine (DAB) substrate kit (Zhongshan Golden Bridge, Beijing, China), and counterstained with hematoxylin. The tissue sections were observed and captured with a microscope.

### Cell culture, in vitro decidualization

Endometrial stromal cell line (T-HESCs) were purchased from American Type Culture Collection (LGC Standards, Milan, Italy). Cells were cultured in maintenance medium of DMEM (Thermo Fisher Scientific, Waltham, MA, USA) with 10 % FBS (Sigma-Aldrich, St. Louis, MO, USA).

For decidualization, cells were grown in 2 % charcoal/dextran treated FBS medium with or without 1 µM MPA and 0.5 mM 8-Br-cAMP, according to the previous methods [19]. After 1, 2, 4, and 6 days incubation, the decidualized cells were harvested. To check for the status of decidualization, cell morphology was photographed by an Axiovert 200 inverted microscope (Zeiss) and the mRNA levels of *IGFBP1* (insulin-like growth factor binding protein 1) and *PRL* (prolactin) were measured by qRT-PCR.

### Vectors construction and lentiviruses infection

To construct hsa_circ_001946 overexpressing vector (hsa_circ_001946), Genomic DNA from 293T cells served as a template for amplification of the full length of hsa_circ_001946 and miR-135b, respectively and the products were inserted in the pLC5-ciR vector (Genessed Biotech, Guangzhou, China) and pCDH-CMV-MCS-EF1-copGFP-T2A-puro. The pLC5-has_circ_0001946 vectors were amplified and cotransfected with psPAX2 and pMD2.G (GenePharma) into 293T cells using transfection reagent Calcium Phosphate Transfection Kit (Invitrogen, USA) to gain infectious lentiviruses LV-has_circ_0001946. The pCDH-miR-135b vectors were amplified and cotransfected with pCMV-Δ8.2, and pCMV-VSV-G into 293T cells using Calcium Phosphate Transfection Kit to gain infectious lentiviruses LV-miR-135b. These recombinant lentiviruses were used to infect T-HESCs to gain cells overexpressing has_circ_0001946 or miR-135b.

### Quantitative RT-PCR

Total RNA was extracted from biopsied endometrial tissues and T-HESCs using Trizol reagent (Invitrogen, Carlsbad, CA, USA) in accordance with the producer’s instructions. The RNA quantity and quality were assessed through NanoDrop-2000 spectrophotometry (Thermo Fisher Scientific, Inc.) at 260 and 280 nm wavelengths. Quality samples had an A260/280 value between 1.8 and 2.1. Complementary DNA (cDNA) was synthesis using SuperScript III kit (Invitrogen/ Life Technologies). The qRT-PCR was performed using the Hieff™ qPCR SYBR® Green Master Mix (YEASEN, Shanghai, China). Reactions were performed in triplicate. The relative expression level was presented using the 2^−ΔΔCt^ formula, with *U6* and *GAPDH* as the endogenous controls, respectively. Primer sequences used in the study were listed in Table 1.

**Table 1:** Primer sequences were used in the study

Primers	Sequences (5’-3’)
miR-135b-F	CGCGTATGGCTTTTCATTC
miR-135b-R	AGTGCAGGGTCCGAGGTATT
U6-F	CTCGCTTCGGCAGCACA
U6-R	AACGCTTCACGAATTTGCGT
GAPDH-F	TCAAGATCATCAGCAATGCC
GAPDH-R	CGATACCAAAGTTGTCATGGA
hsa_circ_0001946-F	TTGGGTCTGTCAGTGGACAA
hsa_circ_0001946-R	GTCACACACCCACCACATTTC
HOXA10-F	GGATTCCCTGGGCAATTCCA
HOXA10-R	CTAATCTCTAGGCGCCGCTC
PRL-F	AGGATCGCCATGGAAAGGGT
PRL-R	GTCAAACAGGTCTCGAAGGGT
IGFBP1-F	TGGGACGCCATCAGTACCTA
IGFBP1-R	CTCCTGATGTCTCCTGTGCC

### Western blotting

Cells were lysed using RIPA buffer (Beyotime, Shanghai, China) supplemented with protease inhibitor cocktail and phosphatase inhibitor cocktails 2 and 3 (Sigma, St Louis, MO, USA). Protein levels were measured using Micro BCA protein assay kit (Pierce, Rockford, IL, USA). Equal amount of protein was separated using 10 % sodium dodecyl sulphate-polyacrylamide gel electrophoresis. Separated proteins were then transferred onto polyvinylidene fluoride (PVDF; Roche, Basel, Switzerland)) membrane using wet transfer method. After blocking in 5 % skim milk at room temperature for 1 h, the membrane was cut into protein bands and incubated with corresponding primary antibodies overnight at 4 °C. The used primary antibodies were anti-HOXA10 (concentration: 0.4 µg/ml; Abcam; ab191470), CCNB1 (dilution: 1:1000; Cell Signaling Technology, Danvers, MA, USA), CDK2 (dilution: 1:1000; Cell Signaling Technology; #2546), CCND1 (dilution: 1:1000; Cell Signaling Technology; #2922), β-actin (dilution: 1:1000; Cell Signaling Technology; #4970). Subsequently, the proteins were incubated with appropriate horse-radish peroxidase (HRP)-conjugated secondary antibody (dilution: 1:2000; Cell Signaling Technology; #5571) at room temperature for 1 h. Then the protein bands were detected using enhanced chemiluminescence kit (Beyotime, Shanghai, China) with a BioSpectrum Imaging System (Bio-Rad, CA, USA).

### Dual Luciferase reporter assay

Possible hsa_circ_001946 binding sequences for miR-135b and miR-135b binding sequences for HOXA10 were predicted using circinteractome and TargetScan. The miR-135b mimics (miR-135b) and miR-NC for transfection experiments were designed and synthesized by RiboBio (Guangzhou, China). The hsa_circ_0001946 or HOXA10 sequences were inserted into pmiR-GLO vector- (Promega, Madison, USA) to product reporter plasmids (hsa_circ_0001946-WT, hsa_circ_0001946-MUT, HOXA10-WT or HOXA10-MUT plasmids). For luciferase reporter assay examining the interaction between miR-135b and hsa_circ_001946, the reporter plasmids ((hsa_circ_0001946-WT or hsa_circ_0001946-MUT) with either miR-135b or miR-NC were co-transfected into T-HESCs using Lipofectamine 2000 (Invitrogen, USA). For luciferase reporter assay examining the interaction between hsa_circ_001946 and HoxA10, reporter plasmids (HOXA10-WT or HOXA10-MUT plasmids) with the miR-135b, miR-NC, miR-135b + vector, or miR-135b + hsa_circ_001946 were transfected into T-HESCs. Following 48 hours’ incubation, the firefly and renilla fluorescence values were detected. The results were presented as the ratio of the two florescence values, with the fluorescence value of renilla plasmid being used as the denominator.

### Cell proliferation assay

Cell proliferation was assessed using 3-(4,5-dimethylthiazol-2-yl)-2, 5-diphenyl tetrazolium bromide (MTT) assay Kit (Cayman, USA). Cells were plated in a 96-well plate at a concentration of 3 ⋅ 10^4^ cells/ well with complete medium. At 0, 24, 48, 72 h, 5 mg/mL of pre-configured MTT reagent was added to the medium, followed by 4 hours’ incubation at 37 °C. After adding 100 µL of dissolving solution (DMSO) to each well, cells were incubated in the dark for 5 min with low speed shake. Optical density (O.D.) was read on a multiwell scanning spectrophotometer at 490 nm.

### Flow cytometry analyses of cell cycle

Cell cycle distribution was further evaluated using cell cycle staining Kit (Liankebio, Hangzhou, China) on BD Biosciences LSRII flow cytometer (BD Biosciences, San Diego, CA). Results were analyzed by the use of FlowJo software (FlowJo, Ashland, USA).

### Data analysis

All results were from three independent experiments and were processed using GraphPad Prism 7 software (GraphPad Software, Inc, La Jolla, CA, USA). Comparison between groups was analyzed using Student’s T-test and differences between more than two groups were analyzed using one-way analysis of variance. *P* value < 0.05 was considered statistically significant.

## Results

### Hsa_circ_001946 and HOXA10 were down-regulated, and miR-135b was up-regulated, in endometrial tissues of women with recurrent implantation failure

**Fig. 1:**
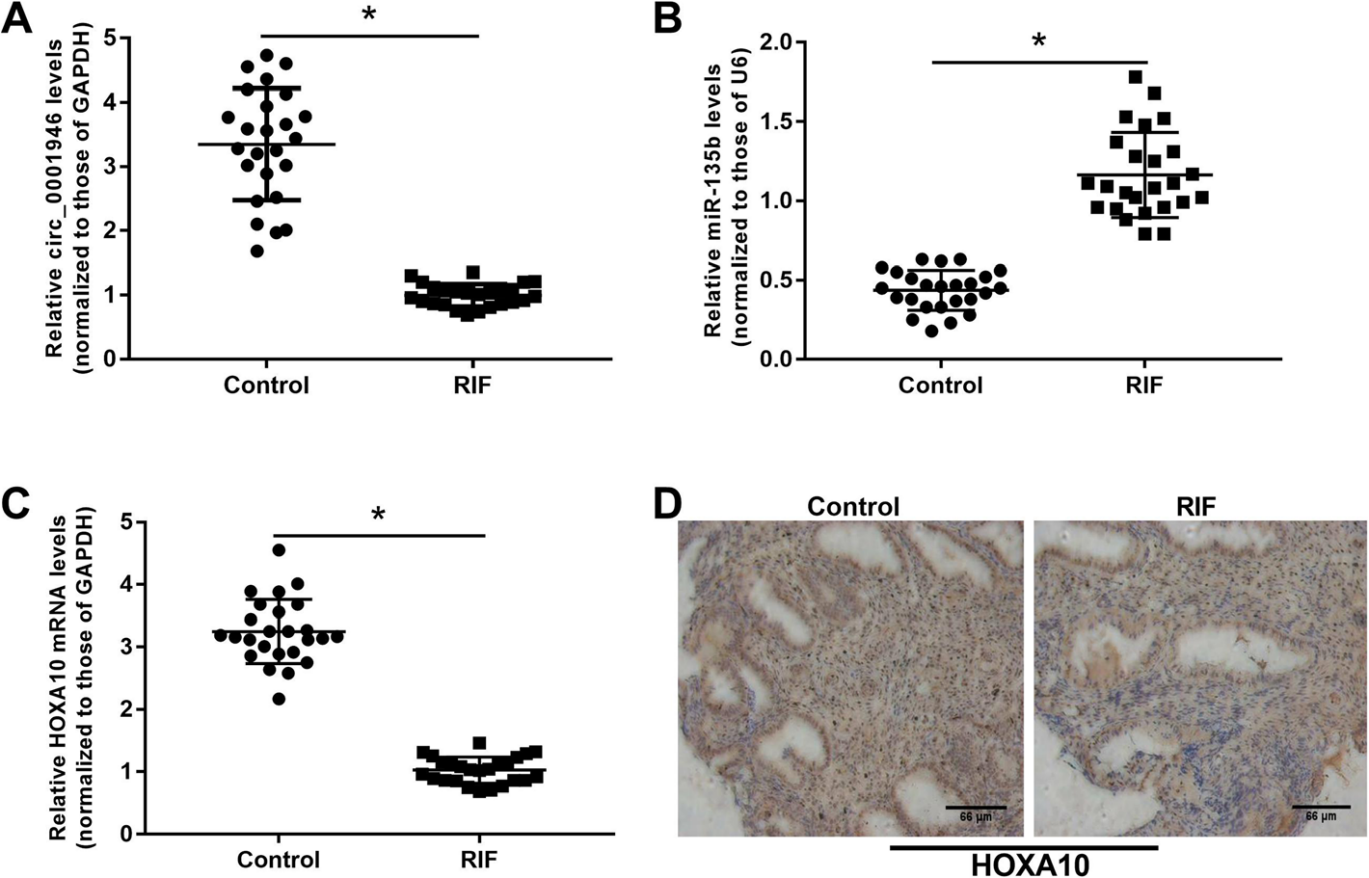
Expression profiles of hsa_circ-0001946, miR-135b, and HOXA10 in tissues from women undergoing ***in vitro*** fertilization-embryo transfer treatment.** a-c.** Quantitative RT-PCR for the expression of hsa_circ_001946, miR-135b, and HOXA10 in tissues from women undergoing *in vitro* fertilization-embryo transfer treatment. **d**. IHC assays for HOXA10 expression. ^*^P < 0.05

First, expression of hsa_circ_001946, miR-135b, and HOXA10 was determined in biopsied endometrial tissues from women undergoing *in vitro* fertilization by qRT-PCR. A significant decrease in hsa_circ_001946 expression (Fig. 1 a), a significant increase in miR-135b expression (Fig. 1b), and a significant decrease in HOXA10 expression (Fig. 1 c and d) were observed in tissues from women with recurrent implantation failure comparing with women who conceived after their implantation.

### Hsa_circ_001946 positively regulates HOXA10 by sponging miR-135b in T-HESCs

**Fig. 2:**
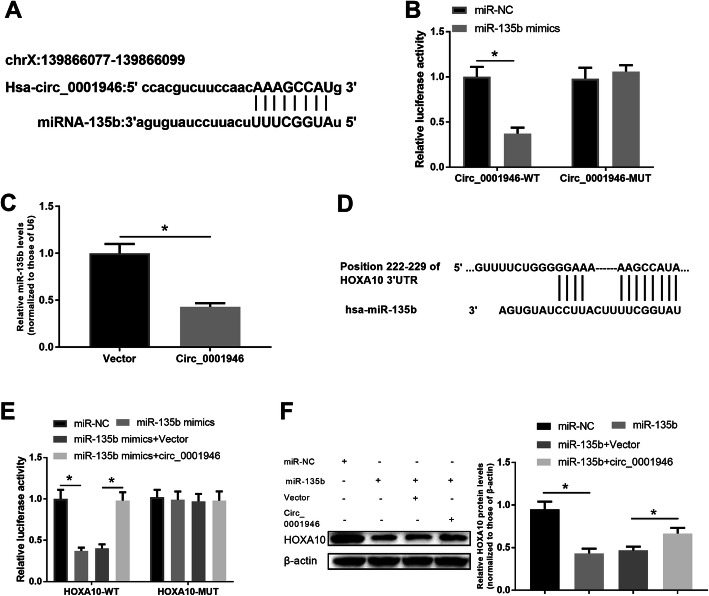
Hsa_circ_001946 functions as a sponge of miR-135b and HOXA10 is a target gene of miR-135b in T-HESCs.** a.** Predicted binding sites for miR-135b on hsa_circ_001946 by circinteractome. **b.** Dual luciferase reporter assays for interaction between hsa_circ_001946 and miR-135b. **c**. Quantitative RT-PCR analyses for expression of miR-135b in T-HESCs transfected with hsa_circ-001946. **d.** Predicted binding sites for miR-135b on the 3’UTR of HOXA10 by TargetScan. **e**. Dual luciferase reporter assays for interaction among miR-135b, HOXA10 and hsa_circ-001946. **f**. Western blot analyses for the expression of HOXA10 in T-HESCs transfected with hsa_circ_001946 and/or miR-135b. ^*^P < 0.05

MiR-135b was predicted to be a functional target of hsa_circ_001946. Figure 2 a displayed the possible binding sites predicted by bioinformatics website (circinteractome). To verify the prediction, dual-luciferase reporter assays were performed. Compared with miR-NC, transfection with miR-135b significantly reduced the luciferase activity of -hsa_circ_001946-WT but not the luciferase activity of hsa-circ_001946-MUT in T-HESCs (Fig. 2b). Also, overexpression of hsa_circ_001946 obviously suppressed the expression of miR-135b in T-HESCs (Fig. 2 c). Recently, studies have reported that miR-135b regulates endometrial receptivity by suppressing HOXA10 [6, 7], but the mechanism for this regulation has not been well examined. Computational prediction by TargetScan identified the potential binding sites for miR-135b on 3’UTR of HOXA10 mRNA (Fig. 2d). As a competing endogenous RNA for miR-135b, hsa_circ_001946 may involve in regulating HOXA10 via sponging miR-135b. Transfection with miR-135b markedly reduced the luciferase activity of the HoxA10-WT compared with miR-NC, and the reduced activity was restored by overexpressing hsa_circ_001946 (Fig. 2e). Luciferase activity of the HoxA10-MUT did not differ among treatments (Fig. 2e). Also, the levels of HOXA10 protein were significantly inhibited when transfected with miR-135b and the decrease was reversed by transfecting with hsa_circ_001946 (Fig. 2 f). These results suggested that hsa_circ_001946 can sponge miR-135b and then promote HOXA10 expression in T-HESCs.

### Hsa_circ_001946 promoted cell proliferation and increased the number of cells at S stage by sponging miR-135b and increasing HoxA10 expression in T-HESCs

**Fig. 3:**
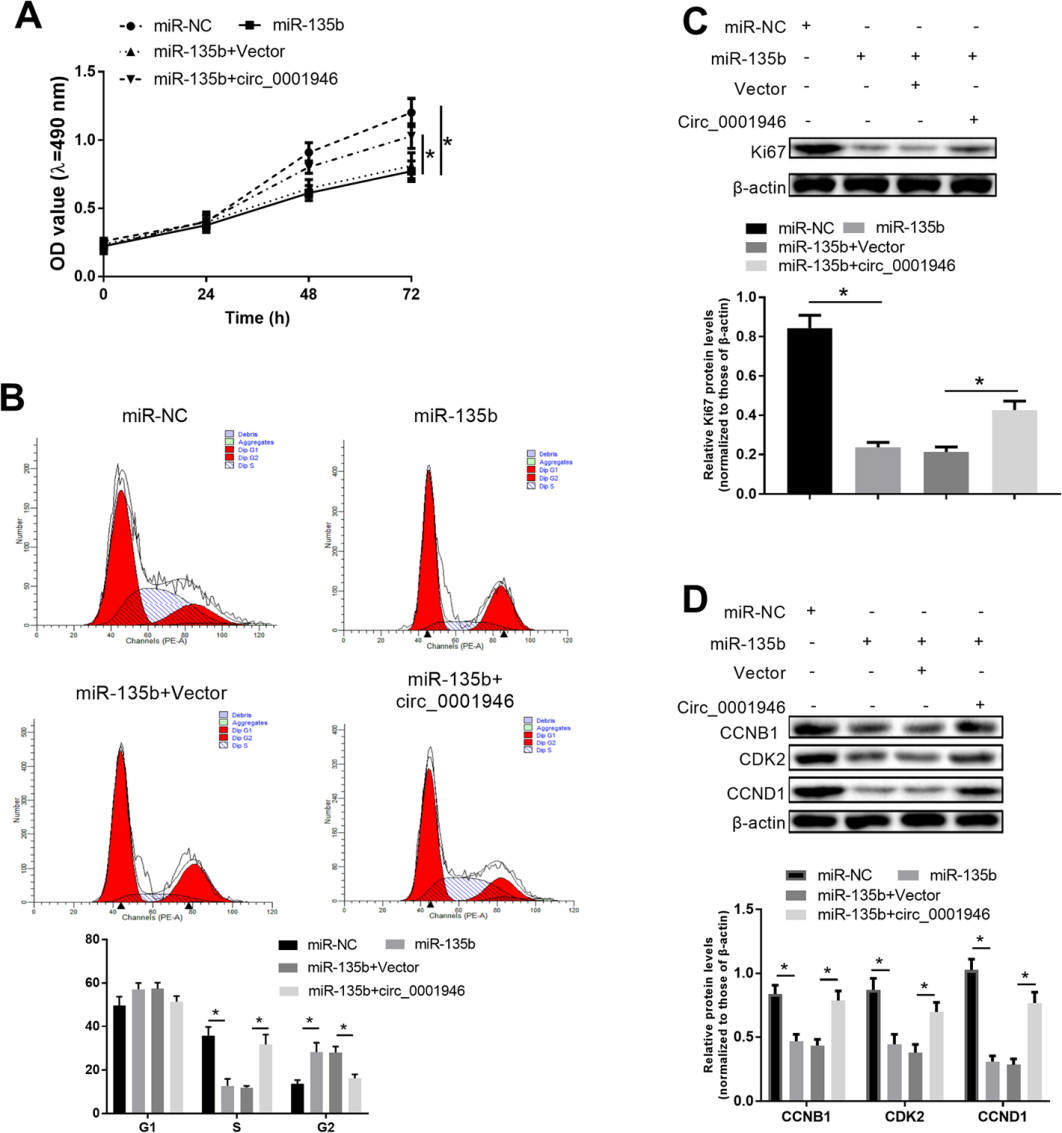
Hsa_circ_001946 promoted proliferation and cell cycle progression by sponging miR-135b in T-HESCs. Cells were overexpressed with hsa_circ_001946 and/or miR-135b. **a.** MTT for proliferation. **b.** Flow cytometry for cell cycle. **c** and **d**. Western blot assays for detecting the expression of Ki67, CCNB1, CDK2, and CCND1. ^*^P < 0.05

In order to clarify the roles and mechanisms of hsa_circ_001946 on endometrial receptivity, proliferation assays and cell cycle assays were performed on T-HESCs transfected with miR-NC, miR-135b, miR-135b + hsa_circ_001946, or miR-135b + vector. MTT assays showed that transfection with miR-135b significantly inhibited cell proliferation, while transfection with hsa_circ_001946 abolished this inhibition (Fig. 3 a). Cell cycle assays indicated that overexpression of miR-135b decreased the number of cells at S stage and increased the number of cells at G2 stage (Fig. 3b). The alterations in cell cycle by miR-135b were reversed by overexpressing hsa_circ_001946 (Fig. 3b). Furthermore, overexpression of miR-135b obviously lowered the protein levels of cell proliferation marker (Ki67) and cell cycle markers (CCNB1, CDK2, and CCND1), which reversed by the overexpression of hsa_circ_001946 (Fig. 3 c and d). Together, hsa_circ_001946 promoted proliferation and advanced the cell cycle process in T-HESCs by competitively binding to miR-135b and increasing the expression of HOXA10.

### hsa_circ_001946 was elevated, miR-135b was reduced, and HOXA10 was elevated in the decidualized T-HESCs

**Fig. 4:**
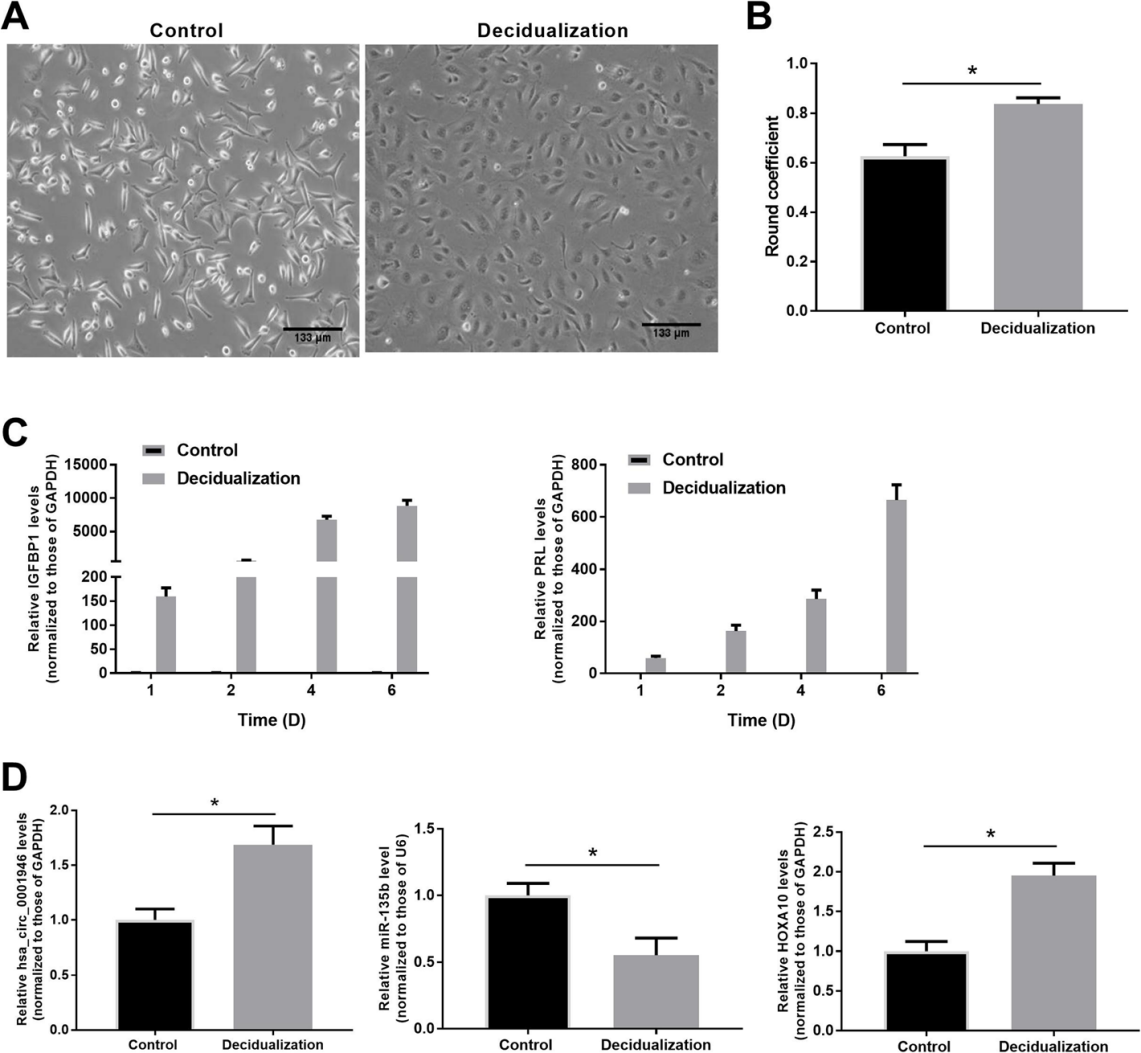
Hsa_circ_001946 was elevated, miR-135b was decreased, and HOXA10 was elevated in decidual T-HESCs. T-HESCs were decidualized with 1 µM MPA and 0.5 mM 8-Br-cAMP hormone treatment. **a**. Cell morphology of T-HESCs under microscope. **b.** Image J software was used to analyze roundness coefficient of T-HESCs. **c.** Quantitative PCR for key decidualization related markers (IGFBP1 and PRL) in T-HESCs. **d**. Quantitative RT-PCR analyses for expression of hsa_circ_001946, miR-135b and HOXA10 in cells. ^*^P < 0.05

Decidualization is the process that transforming stromal fibroblasts into specialized secretory cells, which is the foundation of establishing a receptive endometrial microenvironment to support and maintain pregnancy. To explicate the role of hsa_circ_001946 in endometrial receptivity, we performed *in vitro* decidualization of T-HESCs by MPA and 8-Br-cAMP hormone treatment and then evaluated the expression pattern of decidual markers, *IGFBP1* (insulin-like growth factor-binding protein 1) and *PRL* (prolactin) in the treated T-HESCs. As shown in Fig. 4 a, the untreated T-HESCs had a spindle-shaped fibroblast-like appearance via light microscopy, while treated cells presented a typical morphology of decidual cells characterized by the presence of larger and rounder cells with larger nuclei and abundant cytoplasm. In Fig. 4b, Roundness analysis showed that the rounding of treated cells was significantly increased. Also, the expression of IGFBP1 at days 4 and 6 was markedly elevated and PRL expression levels at days 2, 4 and 6 were significantly elevated in decidualized T-HESCs compared with untreated T-HESCs (Fig. 4 c). Further, expression levels of hsa_circ_001946, miR-135b, and HOXA10 in the decidualized cells were detected. qRT-PCR results revealed that expression of hsa_circ_001946 was elevated, miR-135b was reduced, and HOXA10 was elevated in the decidualized T-HESCs compared to the untreated T-HESCs (Fig. 4d). These results implied that the abnormal expression of hsa_circ_001946, miR-135b, and HOXA10 might be involved in the process of T-HESCs decidualization *in vitro*.

### Overexpression of hsa_circ_001946 promoted decidualization in T-HESCs by sponging miR-135b

**Fig. 5:**
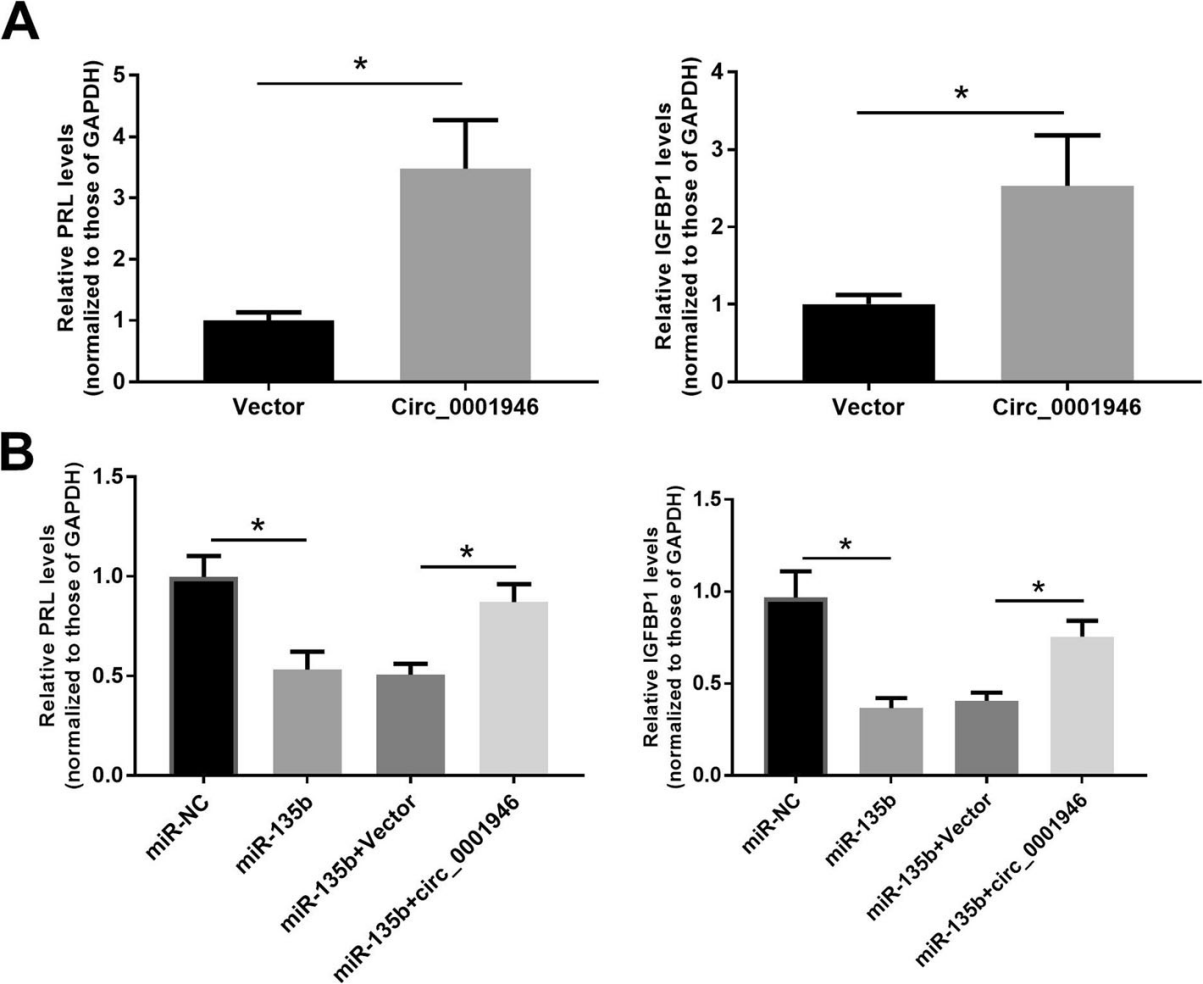
Hsa_circ_001946 promoted decidualization by sponging miR-135b in T-HESCs. Cells were transfected with hsa_circ_001946 and/ or miR-135b. **a **and **b.** Quantitative RT-PCR for IGFBP1 and PRL in the transfected cells. ^*^P < 0.05

Further, to explore the roles of hsa_circ_001946 and miR-135b in the process of T-HESCs decidualization, *in vitro* T-HESCs cells were transfected with hsa_circ_001946, Vector, miR-NC, miR-135b, miR-135b + Vector, or miR-135b + hsa_circ_001946. qRT-PCR results showed that overexpressing hsa_circ-001946 elevated, whereas overexpressing miR-135b suppressed the expression of *PRL* and *IGFBP1* compared with their respective control (Fig. 5 A). Moreover, miR-135b upregulation significantly inhibited the expression of *PRL* and IGFBP1, and the suppression of *PRL* and *IGFBP1* by miR-135b upregulation was reversed by hsa_circ_001946 in T-HESCs (Fig. 5B). All above results suggest that hsa_circ_001946 sponges miR-135b and then enhances the expression of HOXA10 to positively modulate decidualization in T-HESCs.

## Discussion

Implantation failure has been an unsolved impediment for *in vitro* fertilization-embryo transfer, which upsets both the patients and the therapy producers. Receptive endometrium is a premise for successful implantation [20]. Endometrial receptivity is a limited time-frame during which the endometrium is favorable for embryo gets adhered to the luminal epithelium, and further implanted onto the endometrium [4]. To achieve endometrial receptivity, endometrial stromal cells need to under rapid proliferation, followed by a process of differentiation (decidualization) which transforms proliferating fibroblasts to secretory cells capable of producing factors that facilitate the implantation.

The hyperproliferation and the decidualization are under a regulation of a complicated succession of genetic and epigenetic changes [21]. CircRNAs are a new class of non-coding RNAs, whose field of research has been boomed recently. Recently, Shen J and his colleagues have determined that acupuncture and moxibustion have effects on endometrial receptivity via modulating circRNAs associated with infertility [22]. Emerging evidences show that circRNAs can act as a sponge of microRNAs and up-regulate the target mRNAs [13]. It has been suggested that circBACH1 may function as a ceRNA of miRs to influence endometrial receptivity [22]. In dairy goats, Circ-8073 has been determined to be involved in the development of endometrial receptivity by increasing CEP55 expression and elevating proliferation of endometrial epithelium cells by sponging miR-449a [15]. MicroRNA -135b is a microRNA whose expression is closely related with the receptivity status of endometrium via downregulating HOXA10, a receptivity marker [7, 23]. CircRNA_0001946, also known as CDR1as and CiRS-7, is derived from chrX: 139,865,339–139,866,824 [24]. Numerous studies have reported that circ_001946 is abnormally upregulated and knockdown of its expression represses cancer cell growth and migration [25, 26]. To our best knowledge, no investigation has been conducted regarding the role of hsa_circ_001946 on endometrial receptivity. We collected endometrial tissues from 50 women undergoing *in vitro* fertilization process in our center, with 25 suffering recurrent implantation failure and 25 successfully conceived after implantation. Quantitative PCR analyses showed a marked reduction of hsa_circ_001946 expression, a significant elevation of miR-135b expression, and an obvious reduction of HOXA10 in the tissues from women patients suffering recurrent implantation failure. The differences in the expression of miR-135b and HOXA10 were in concordance with prior studies [6, 7], and what we observed suggested there might be an association among hsa_circ_001946, miR-135b, and HOXA10. Thus, in the present investigation, we focused on the roles and molecular mechanism of hsa_circ_001946, miR-135b, and HOXA10 in endometrial receptivity.

A negative effect of miR-135b on HOXA10 and endometrial receptivity has been reported in several studies, but the mechanism for this particular regulation is not well illustrated [6, 7]. To investigate the association among hsa_circ_001946, miR-135b, and HOXA10, we employed online bioinformatics tools (circinteractome and TargetScan). Our results suggested several binding sites of miR-135b on hsa_circ_001946 and the 3’UTR of HOXA10. The predicted interactions were subsequently verified in T-HESCs using dual luciferase reporter assays. Our results revealed that hsa_circ_001946 upregulated the expression of HOXA10 by functioning as a sponge of miR-135b.

The expression of HOXA10 is markedly increased during the mid-secretory phase, which is indispensable for the initiation of the hyperproliferation of endometrial stromal cells [12, 27]. We speculated that hsa_circ_001946 may affect endometrial stromal cell proliferation via regulating the level of HOXA10. Our results showed that overexpression of miR-135b suppressed the proliferation of T-HESCs, whereas co-transfection with hsa_circ_001946 reversed the suppression. To further investigate the roles of hsa_circ_001946 in T-HESCs cell proliferation, cell cycle assay was performed. Overexpression of miR-135b decreased the number of cells at S stage, but increased the number of cells at G2/M. Besides, miR-135b decreased cell proliferation marker (Ki67) and cell cycle markers (CCNB1, CDK2, and CCND1) and these inhibitory effects were reversed by hsa_circ_001946 upregulation. These suggested that increase in miR-135b expression decreased cell proliferation and detained cell cycle by arresting cells at G2/M stage, which is in accordance with existing reports. Prior studies have reported that G0/G1 cells decrease slightly, while cells in G2/M phase increase with HOXA10 knockdown, which indicating an arrest of cell at the G2/M stage [17]. Therefore, we concluded that hsa_circ_001946 promote the proliferation and cell cycle of T-HESCs via acting as a sponge of miR-135b and enhancing HOXA10 expression.

Decidualization is a process initiated by the hyperproliferation of endometrial stromal cells and indispensable for developing endometrial receptivity [28]. In this study, *in vitro* decidualization of T-HESCs was performed using MPA and 8-Br-cAMP hormone treatment and results showed that the status of decidualization was assessed by both the cell morphology and the expression of key decidualization related markers (IGFBP1 and PRL). In the present study, we observed an increase of hsa_circ_001946, a decrease of miR-135b, and an increase in HOXA10 expression in decidualized T-HESCs compared to untreated cells. Previous studies have revealed that miR-135b decreases its expression and HOXA10 raises its expression during the 17b-estradio induces decidualization [23]. Also, the increased levels of miR-135b using miRNAs mimics in T-HESCs under the decidualization treatment numerically reduced the secretion levels of the IGFBP1. In this study, hsa_circ_001946 alone boosted, whereas miR-135b alone suppressed the secretion level of IGFBP1 and PRL. Furthermore, hsa_circ_001946 overexpression reversed the decrease caused by miR-135b.

## Conclusions

Conclusively, we determined that hsa_circ_001946 promoted cell proliferation and cell cycle process via acting as a sponge of miR-135b and elevating the expression of HOXA10 and increased expression of decidualization markers to enhance the development of endometrial receptivity. Our findings may provide a new insight about mechanism of endometrial dysfunction as well as female reproductive failure and suggest that hsa_circ_001946 may be critical for endometrial receptivity and establishment and maintenance of pregnancy.

## Data Availability

The datasets generated during the current study are available from the corresponding author on reasonable request.

## Abbreviations

qRT-PCR: Quantitative RT-PCR
MPA: Medroxyprogesterone acetate
8-Br-cAMP: 8-bromoadenosine 3’:5’-cyclic monophosphate
HOXA10: HomeoboxA-10
circRNAs: Circular RNAs
TBST: Tris-buffered saline, 0.05 % Tween 20
DAB: 3,3′-diaminobenzidine
T-HESCs: Endometrial stromal cell line
IGFBP1: Insulin-like growth factor binding protein 1
PRL: Prolactin
HRP: Horse-radish peroxidase
MTT: 3-(4,5-dimethylthiazol-2-yl)-2, 5-diphenyl tetrazolium bromide
OD: Optical density
